# The Role of the Microbiome in Asthma: The Gut–Lung Axis

**DOI:** 10.3390/ijms20010123

**Published:** 2018-12-30

**Authors:** Franco Frati, Cristina Salvatori, Cristoforo Incorvaia, Alessandro Bellucci, Giuseppe Di Cara, Francesco Marcucci, Susanna Esposito

**Affiliations:** 1 Pediatric Clinic, Department of Surgical and Biomedical Sciences, Università degli Studi di Perugia, 06132 Perugia, Italy; francofrati57@gmail.com (F.F.); crisalva_@libero.it (C.S.); alessandro.bell992@gmail.com (A.B.); giuseppe.dicara@unipg.it (G.D.C.); fmarcucci99@yahoo.it (F.M.); 2 Cardiac/Pulmonary Rehabilitation, ASST G. Pini/CTO, 20122 Milano, Italy; cristoforo.incorvaia@gmail.com

**Keywords:** asthma, atopy, gut microbiota, immunity, lung microbiota, microbiome

## Abstract

Asthma is one of the most common chronic respiratory diseases worldwide. It affects all ages but frequently begins in childhood. Initiation and exacerbations may depend on individual susceptibility, viral infections, allergen exposure, tobacco smoke exposure, and outdoor air pollution. The aim of this review was to analyze the role of the gut–lung axis in asthma development, considering all asthma phenotypes, and to evaluate whether microbe-based therapies may be used for asthma prevention. Several studies have confirmed the role of microbiota in the regulation of immune function and the development of atopy and asthma. These clinical conditions have apparent roots in an insufficiency of early life exposure to the diverse environmental microbiota necessary to ensure colonization of the gastrointestinal and/or respiratory tracts. Commensal microbes are necessary for the induction of a balanced, tolerogenic immune system. The identification of commensal bacteria in both the gastroenteric and respiratory tracts could be an innovative and important issue. In conclusion, the function of microbiota in healthy immune response is generally acknowledged, and gut dysbacteriosis might result in chronic inflammatory respiratory disorders, particularly asthma. Further investigations are needed to improve our understanding of the role of the microbiome in inflammation and its influence on important risk factors for asthma, including tobacco smoke and host genetic features.

## 1. Introduction

Asthma is one of the most common chronic respiratory diseases worldwide. It affects all ages but frequently begins in childhood. It is a chronic inflammatory disorder of the lower airways that is characterized by wheezing, shortness of breath, chest tightness, and cough, which may vary over time in their occurrence, frequency and severity [1]. The symptoms are associated with variable expiratory airflow impairment, i.e., breathing difficulty with prolonged expiration due to bronchoconstriction (airway narrowing), airway wall thickening, and increased mucous production. Epidemiological studies have estimated that 250,000 deaths can be linked to this disease each year, and more than 600 million people have asthma-related symptoms [1]. A new approach was recently developed using a standardized unit, called “disability-adjusted life year” (DALY). DALY assesses the years of healthy life lost because of a disease, combining information about morbidity and mortality in terms of healthy years lost. Worldwide, asthma accounts for nearly 1% of all DALYs lost, resulting in a particularly high morbidity [2].

Asthma is a complex disease that includes multiple phenotypes with diverging clinical and pathophysiological characteristics [3,4]. Asthma initiation and exacerbation may depend on individual susceptibility, viral infections, allergen exposure, tobacco smoke exposure, and outdoor air pollution [3,4]. If one considers all allergic inflammation triggers, the role of environmental allergen exposure is very relevant. The most common allergens involved in asthma development and exacerbation are dust mites, grass, tree pollen, animal epithelia, fungi, and mold [3,4]. The incidence of allergic diseases has increased dramatically over the last five decades, with large variations in the prevalence of asthma in different countries. Although asthma symptoms are generally more common in some high-income countries, several low- and middle-income countries also show high levels of asthma prevalence [5]. Among children, asthma is usually more severe in low- and middle-income than high-income countries [5].

The decreasing number of infections in Western countries, and more recently in developing countries, seems to be at the origin of the increasing incidence of both autoimmune and allergic diseases [6]. The underlying mechanisms of this event are multiple and complex, involving various regulatory T cell subsets and Toll-like receptors (TLRs). These mechanisms could derive, to some extent, from changes in the microbiota caused by changes in lifestyle. The “hygiene hypothesis” was the first to suggest a link between microbes and allergy [7,8]. Recently, the original concept of the hygiene hypothesis was expanded to include the increase of antibiotic use and vaccinations, as other lifestyle changes have reduced childhood infections and altered the microbiota [9]. Moreover, other important perinatal and early postnatal factors include caesarean birth and milk formula feeding [9]. Another significant issue is the change in the modern diet based on high levels of fat and low levels of fiber, which has profound consequences for the composition of the intestinal microbiome [10,11,12,13,14,15,16].

Interestingly, an important study by Stein et al. underlined the importance of living in a healthy environment for asthmatic patients, especially near dairy farms [17]. The study evaluated 60 Amish and Hutterite children; levels of allergens and endotoxins were measured, and an assessment of the microbiome composition of indoor dust samples was performed. These U.S. farming populations have a similar lifestyle but different agricultural practices. In fact, the Amish use traditional farming, while Hutterites make use of mechanized techniques. Albeit comparable in genetic heritage and standard of living, it was found that in the Amish the prevalence of asthma and allergies is four and six times lower than in Hutterites, respectively. This is associated with the occurrence in Amish dwellings of average endotoxin levels approximately seven times higher. Analyzing dust samples from Amish and Hutterite houses, differences in microbial presence were detected. In addition, in two groups of Amish and Hutterite children, significant differences were observed concerning innate immune cells in quantitative and functional terms, as well as in phenotypes [17]. The conclusion was that the Amish environment provides protection against asthma by engaging and shaping the innate immune response. The aim of this review was to analyze the role of the gut–lung axis in asthma development considering all asthma phenotypes and to evaluate whether microbe-based therapies may be used for asthma prevention.

## 2. Microbiome and Atopy

Atopy can be defined as a genetic disposition to develop an allergic reaction and produce elevated levels of IgE upon exposure to an environmental antigen and especially one inhaled or ingested [18]. The chronic inflammation of atopic disease is caused by an enhanced T-helper 2 (Th2)-type immune response against common, non-pathogenic, environmental antigens (i.e., allergens) in susceptible individuals because of their genetic background [18,19]. Th cells play an important role in immune system regulation: Th2 and Th17 cells in particular direct immune response and coordinate other cells (i.e., B cells, eosinophils, mast cells, or neutrophils). Th2 cells produce various cytokines, such as interleukin (IL)-4, IL-5, IL-9, IL-10, and IL-13. Furthermore, IL-4 stimulates B cells to produce eosinophils and IgE antibodies, which in turn enhance mast cells to release histamine, serotonin, and leukotrienes to cause bronchoconstriction, contributing to allergic responses. The differentiation of naive T cells into IL-4-secreting T cells is one of the hallmarks of allergy [18,19].

A potential role of the gut microbiota in atopy is based on the observation of germ-free mice, which are born and raised in a sterile environment. They are more susceptible to anaphylaxis induced by the administration of oral antigens when compared to mice that are not germ-free, demonstrating how difficult it is to achieve oral tolerance in animals with altered microbiota [20,21]. To induce and maintain oral tolerance, the role of regulatory T cells (Tregs) is fundamental. Tregs are a subpopulation of T cells that modulate immune system activity, maintain tolerance to self-antigens, and prevent autoimmune disease development. In recent years, it has also become clear that the induction of Tregs is influenced by symbiotic microbes and may therefore provide a possible link between our environment and the susceptibility to allergic disorders [18]. Several studies in the last 10 years have confirmed a role for the microbiota in regulating immune function. For example, *Bacteroides fragilis* modulates the Th type 1/2 (Th1/Th2) balance [22], and segmented filamentous bacteria directly stimulate Th17 cell differentiation [23], whereas *Clostridium* spp. induce Treg production [24]. Furthermore, the microbiome produces several mediators, such as lipopolysaccharides (LPS), peptidoglycans, short-chain fatty acids (SCFAs), and gaseous molecules, which influence host physiology depending upon the dose, developmental time period, and tissue type [25,26,27,28,29]. Furthermore, the administration of LPS to germ-free animals was sufficient to restore oral tolerance [30]. For example, *Clostridium* spp. are producers of propionic acid (PPA) following the fermentation of complex carbohydrate fibers that are implicated in the modulation of cell signaling, immune function (generation of Tregs), and neurotransmitter synthesis and release [27,28]. Another important metabolite that depends on microbiota metabolism is tryptophan, which can regulate serotonin production and induce many significant effects on brain function, contributing to psychiatric diseases [31,32,33].

The innate immune response is based on a number of defensive systems providing nonspecific reactions to environmental stimuli. Such responses include both cellular and soluble factors. In addition to their role as a mechanical barrier, the airway epithelium and mucosal stratum guarantees innate immunity provided by immune cells such as dendritic cells, various types of innate lymphoid cells (ILCs), as well as different types of leukocytes, including neutrophils, eosinophils, and macrophages [34]. Different ligand motifs are recognized by receptors such as retinoic acid inducible gene I (RIG-I)-like receptors, TLRs, and nucleotide-binding oligomerization domain (NOD)-like receptors. These receptors are important in facilitating the timely responses that result in the ensuing adaptive immunity [35]. The host defense also includes several noncellular secreted elements with antimicrobial action, comprising defensins, lactoferrin, interferons, cathelicidin (LL37), and leukocyte protease inhibitor (SLPI) [36].

## 3. Microbiome and Asthma

Asthma has been extensively studied concerning the innate and adaptive immune response. The continuous exposure of the respiratory system to airborne agents in patients with a genetic predisposition offers repeated opportunities for contact with mucosal immunity of inhaled material from upper airways or from the digestive tract, which may contain microbes and bile salts and also provide immune stimuli [33]. The epithelial mucosa and the dendritic cells, which are in continuous contact with the airway lumen, and antimicrobial peptides produced by immune cells play an important role in the response to environmental agents [37]. The epithelium controls the local immune activities of IgA antibodies, defensins, and lysozymes, which are also regulated by the production of IL-25, IL-33, and thymic stromal lymphopoietin (TSLP), that in turn stimulate a Th2 type inflammation, which is known to favor the development of asthma. The relevance of Th2 responses driven by the epithelium was supported in a recent study investigating the effect of treatment with an anti-TSLP antibody, which reduced allergen-induced bronchoconstriction and diminished the markers of Th2-related airway inflammation, including the level of exhaled nitric oxide and the number of eosinophils in the sputum [38]. Epithelial cells also produce other regulatory cytokines, such as IL-10 and transforming growth factor (TGF)-beta. This makes it apparent that the airway epithelium has an important role in orchestrating both the innate and adaptive immunity that is involved in the development of asthma.

Airway mucus, in addition to the known mucociliary clearance, has other key properties of importance in asthma [39]. Mucus contains both aqueous and non-aqueous constituents. Mucins (MUCs) are large glycoproteins, predominant types in human airways being MUC5AC and MUC5B. Experimental data from mice suggest a critical role in protection of airways for MUC5B, but not for MUC5AC. In fact, the lack of MUC5B resulted in lung inflammation, weakened immune homeostasis, and infections from several bacterial species [40]. Proteins with antimicrobial activity are naturally occurring in airway mucus and may exert their action once microbes are trapped in mucus but incompletely eliminated by mucociliary clearance. This is true for cystic fibrosis (CF), wherein repeated chronic infection from *Pseudomonas aeruginosa* is commonly observed. As opposed to the *P. aeruginosa* phenotype that causes acute infections, in chronic *P. aeruginosa* infection in CF, a noninvasive phenotype with high resistance to eradication prevails. It was recently suggested that in CF the mucus, as well as the high levels of neutrophil elastase, may favor the growth of *P. aeruginosa* aggregates resistant to antibiotics [41]. A further mucous–microbial relation concerns bacteriophages, which are viruses infecting bacterial species. Bacteriophages have the ability to bind to mucin glycans, thus reducing bacterial adherence to epithelial cells of the airways [42]. This adherence has an important role because it may be a form of protection from bacterial infections that are non-host-derived.

An important link between the innate and adaptive immunity is represented by dendritic cells (DCs), which influence the response to virus infection and the development of allergic inflammation [43]. DCs present to the immune system fragments of microbes that promote a number of regulatory and adaptive responses, including Th1, Th2, and Th17 pathways. The role of the lung DCs in asthma was recently studied [44]. DCs may activate several immune cell types, such as T cells and ILCs, the latter comprising a group of cells occurring at barrier surfaces, which are able to provide environmental signals that also target commensal bacteria.

The human gut in otherwise healthy subjects is colonized by 10^14^ bacteria and contains more than 1000 bacterial species [45]. The interaction of the human gut and its microbiome has been conditioned by dietary and environmental variations, leading to the selection of a high variety of bacteria interacting both for defense and nutritional advantages. The microbiome is present in both the placenta and meconium, suggesting that the fetus is exposed to bacteria in the prenatal period [46]. The time and mode of childbirth, maternal age, diet, hospitalization, body mass index, smoking status, socioeconomic status, breastfeeding, and antibiotic use all influence the development of the infant microbiome. Long-term stability for many microbiome species begins at approximately two years of age [47,48,49,50].

For several years, the respiratory tract was considered germ-free, but this belief, which was due to difficulties in bacterial culturing, was recently negated by metagenomic data analysis that revealed the presence of a microbiome at this site also in healthy neonates. Although the gut microbiome contributes to the generation of Tregs and probably influences susceptibility to oral allergens, asthma is thought to originate from inhalation, making the lung (and its microbiome) more relevant for asthma initiation. The differing composition of the lung microbiome between asthmatic and healthy people suggests that bacteria may contribute to the initiation of asthma, also indicating a possible important role in influencing the immune responses for microbiota residing in other sites, such as the gut [51]. This has led to the concept of the “gut–lung axis”. Stiemsma et al. studied a population of children diagnosed with asthma at preschool age in whom they found evidence of gut bacterial dysbiosis [52]. In particular, a reduction of *Lachnospira* in favor of *Clostridium* spp. was potentially linked to asthma. The individual opposing shift in the abundance of *Lachnospira* and *Clostridium neonatale* in the first three months of life suggests that these specific gut bacteria play a role in protecting or promoting development of a preschool age asthmatic phenotype, in addition to the previously identified roles in other atopic disorders. This bacterial dysbiosis was confirmed in other studies of the same group of authors, in which they showed the relative abundance of the bacterial genera *Lachnospira* and the decrease of *Veillonella*, *Faecalibacterium*, and *Rothia* in children at risk of asthma [53]. This reduction was accompanied by decreased levels of faecal acetate and dysregulation of enterohepatic metabolites. Inoculation of germ-free mice with these four bacteria ameliorated airway inflammation [54]. *Clostridium* spp. are implicated in the increased risk of asthma in several other studies [55,56,57].

**Figure 1:**
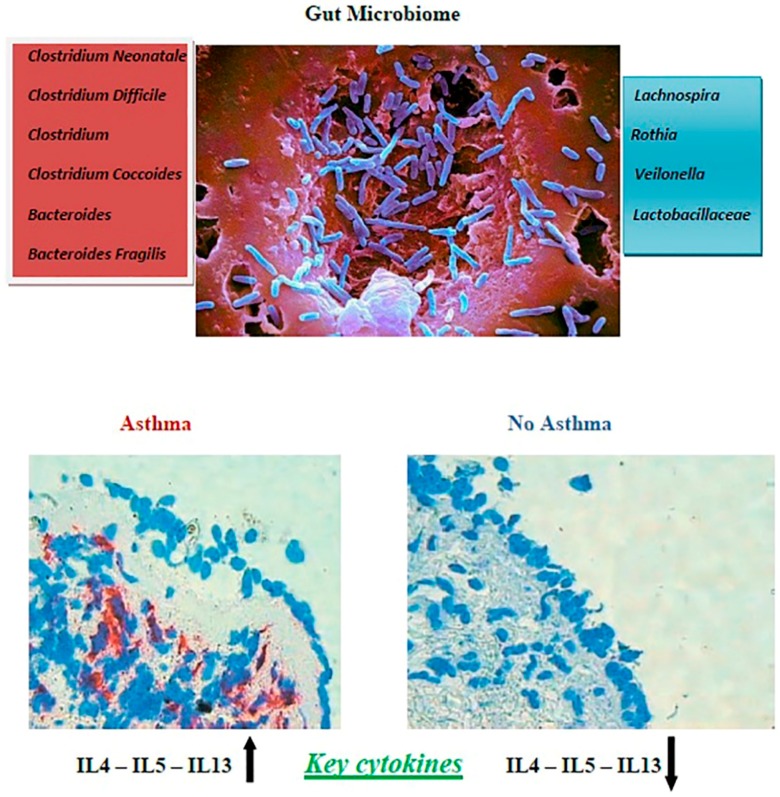
Differences in the lung bacterial microbiota between asthmatic and healthy children. In the upper section, the names and the image of pathogens that can influence asthma development are represented. In the lower section, on the left it is shown the respiratory tract of an asthmatic child with an increase in interleukin (IL)-4, IL-5, and IL-13; on the right is shown the respiratory tract of a healthy child with normal values of IL-4, IL-5, and IL-13.

To underscore the role of mediators produced by the microbiota, another study highlighted the protective effect of a factor called A20 that is expressed by lung epithelial cells. Asthmatic patients showed reduced levels of A20 in epithelial cells, which might make these patients more susceptible to allergic asthma due to failed LPS tolerance induction [58]. The first 100 days of life seem to be the early life “critical window” during which microbial dysbiosis is very influential in promoting the development of IgE-mediated hypersensitivities in humans [59]. From these findings, we can assume that external agents, such as antibiotic or probiotic administration, can modulate the immune response. In experimental models of murine allergic airway disease, Noverr et al. described how antibiotic treatment in immunocompetent hosts followed by oral administration of *Candida albicans* induced susceptibility to allergic airway disease [60,61]. However, oral administrations of *Mycobacterium vaccae* [62], *Helicobacter pylori* [63], as well as conventional probiotic strains [64,65] reduced symptoms of allergic airway disease in mice. One study showed that Tregs produced in the periphery (but not in the thymus) are known as induced Tregs (iTreg) and are principally stimulated in the mesenteric lymph nodes (MLN), Peyer’s patches, and lamina propria (LP) of the small and large intestines [66]. Mice deficient in iTregs spontaneously develop Th2 type pathologies characterized by higher percentages of CD4+ T cells producing IL-4, IL-13, and IL-5 cytokines in the MLN; IL-4 in the LP of the large intestine; and IL-13 and IL-5 in the LP of the small intestine. An enhanced Th2 immune response and the production of cytokines, such as IL-4, IL-13, and IL-5, contribute to the induction of atopic diseases [67]. Figure 1 shows differences in the lung bacterial microbiota between asthmatic and healthy people, showing that the respiratory tract of an asthmatic patient had an increase in IL-4, IL-5, and IL-13 and these differences, together with other environmental factors in individuals with a specific genetic background, may have an impact on the gut microbiome.

Early viral infections, mainly due to rhinovirus or respiratory syncytial virus, are also linked to the development of asthma [68,69]. Moreover, it has been reported that colonization at one month of age with *Streptococcus pneumoniae*, *Moraxella catarrhalis*, or *Haemophilus influenzae* is associated with an increased risk of subsequent asthma development and enhanced Th2-associated immunity [70].

The leading hypotheses to date have been limited to the role of the bacterial and viral microbiota in affecting allergic responses [71,72,73]. Recently, it has been shown that nasal microbiome composition differs in subjects with exacerbated asthma, nonexacerbated asthma, and healthy controls, and nasal taxa could be further investigated as possible biomarkers of asthma activity [74]. Furthermore, it has been observed that one-year-old children with an immature microbial composition have an increased risk of asthma at five years of age [75]. Conversely, adequate maturation of the gut microbiome in this period may protect these pre-disposed children from asthma development [75]. However, in addition to the complex bacterial communities that have been detected in farm dust, fungal loads have also been reported to be high [76]. Within the context of allergies, fungi are typically considered within the framework of allergens or inducers of allergic inflammation [76]. Nevertheless, many factors complicate the analysis of dysbiosis in subjects with food allergy [77]. Comparisons between studies are difficult because of considerable heterogeneity in study design, sample size, age at fecal collection, methods of analysis of gut microbiome, and geographic location. Fungi could also be key regulators of inflammation and immune homeostasis [76,78], opening the avenue of beneficial roles of fungi in shaping immune responsiveness. Indeed, a recent report by Wheeler et al. reported that disruption of the “mycobiome” with an antifungal drug can predispose mice to both colitis and allergic airway inflammation [79].

Interestingly, using advanced multiplex quantitative imaging methods, a recent study has identified an extensive and persistent phosphorylated-STAT3 signature in group 3 ILCs, and intestinal epithelial cells that are induced by IL-23 and IL-22 in mice that lack CD4+ T cells [80]. By contrast, in immune-competent mice, phosphorylated-STAT3 activation is induced only transiently by microbial colonization at weaning. This early signature is extinguished as CD4+ T cell immunity develops in response to the expanding commensal burden. Physiologically, the persistent IL-22 production from group 3 ILCs that occurs in the absence of adaptive CD4+ T cell activity results in impaired host lipid metabolism by decreasing lipid transporter expression in the small bowel. These findings provide new insights into how innate and adaptive lymphocytes operate sequentially and in distinct ways during normal development to establish steady-state commensalism and tissue metabolic homeostasis [80]. Germain’s group showed that ILC2s possess properties considered characteristic of adaptive T lymphocytes, namely local activation and distant effector function, but in response to alarm cytokines instead of specific antigens [81,82]. On the other hand, other authors showed the distinct role of individual commensal bacteria in maintaining immune functions during/following dysbiosis induced by antibiotic therapy, thereby shaping host immunity and opening novel therapeutical avenues in conditions of perturbed microbiota composition [83].

## 4. Conclusions

Recent studies have demonstrated a role for the gut microbiome in influencing remote organs and mucosal and hematopoietic immune functions [84]. The underlying inflammation in atopic asthma seemed related to the composition of microbiota and appeared associated with the severity of airway obstruction [85]. Treatment with inhaled corticosteroids was associated with changes in the airway inflammatory response to microbiota [86,87]. The interaction of different mucosal barriers, including the gut–lung cross-talk, is likely to be mediated by locally resident microbes and circulating immune cells, but further studies are needed to fully understand this issue.

At present, the available treatments for the main non-communicable lung diseases are aimed only at reducing symptoms but are unable to effectively prevent and/or cure the diseases. Recent clinical and basic studies to date have identified possible therapeutics that can target innate immunity and the microbiota in asthma [88]. Studies have examined gut microbiota maturation over the first year of life in infants at high risk for asthma, and whether it is modifiable by early-life *Lactobacillus* supplementation [88]. Results showed that early-life gut microbial development is distinct, but plastic, offering a novel strategy for early-life preventive interventions. Considering the data already collected on the gut–lung axis, manipulation of the airway and gut microbiome, particularly in early life, might be a strategy to prevent asthma initiation and exacerbation. A better understanding of microbiome-driven pathophysiology and inflammation, in conjunction with the interaction of major risk factors for asthma development, such as host genetics and tobacco smoke, would aid in optimizing current treatments and in managing this chronic lung condition.

Furthermore, by improving our understanding of the role of the microbiome in these diseases, novel therapeutic strategies of modifying the microbiome through diet, probiotics, or faecal or selected bacterial transfers may be developed. The effects of these therapies on the overall microbiome and consequently on disease severity/progression remain largely unknown and need to be properly understood to realize the full impact of these treatments. 

## References

[1] Mathew J., Aronow W.S., Chandy D. (2012). Therapeutic options for severe asthma. Arch. Med. Sci..

[2] Masoli M., Fabian D., Holt S., Beasley R. (2004). Global Initiative for Asthma (GINA) Program: The global burden of asthma: Executive summary of the GINA Dissemination Committee report. Allergy.

[3] Forno E., Celedon J.C. (2012). Predicting asthma exacerbations in children. Curr. Opin. Pulm. Med..

[4] Plunkett C.H., Nagler C.R. (2017). The influence of the microbiome on allergic sensitization to food. Immunol.

[5] ISAAC Steering Committee (1998). Worldwide variations in the prevalence of asthma symptoms: The International Study of Asthma and Allergies in Childhood (ISAAC). Eur. Respir. J..

[6] Jatzlauk G., Bartel S., Heine H., Schloter M., Krauss-Etschmann S. (2017). Influences of environmental bacteria and their metabolites on allergies, asthma, and host microbiota. Allergy.

[7] Stiemsma L.T., Turvey S.E. (2017). Asthma and the microbiome: Defining the critical window in early life. Allergy Asthma Clin. Immunol..

[8] Strachan D.P. (1989). Hay fever, hygiene, and household size. BMJ.

[9] Bloomfield S.F., Rook G.A., Scott E.A., Shanahan F., Stanwell-Smith R., Turner P. (2016). Time to abandon the hygiene hypothesis: New perspectives on allergic disease, the human microbiome, infectious disease prevention and the role of targeted hygiene. Perspect. Public Health.

[10] Dominguez-Bello M.G., Costello E.K., Contreras M., Magris M., Hidalgo G., Fierer N., Knight R. (2010). Delivery mode shapes the acquisition and structure of the initial microbiota across multiple body habitats in newborns. Proc. Natl. Acad. Sci. USA.

[11] Bäckhed F., Roswall J., Peng Y., Feng Q., Jia H., Kovatcheva-Datchary P., Li Y., Xia Y., Xie H., Zhong H. (2015). Dynamic and stabilization of the human gut microbiome during the first year of life. Cell Host Microbe.

[12] Guaraldi F., Salvatori G. (2012). Effect of breast and formula feeding on gut microbiota shaping in newborns. Front. Cell. Infect. Microbiol..

[13] David L.A., Maurice C.F., Carmody R.N., Gootenberg D.B., Button J.E., Wolfe B.E., Ling A.V., Devlin A.S., Varma Y., Fischbach M.A. (2014). Diet rapidly and reproducibly alters the human gut microbiome. Nature.

[14] Sonnenburg E.D., Smits S.A., Tikhonov M., Higginbottom S.K., Wingreen N.S., Sonnenburg J.L. (2016). Diet induced extinctions in the gut microbiota compound over generations. Nature.

[15] De Filippo C., Cavalieri D., Di Paola M., Ramazzotti M., Poullet J.B., Massart S., Collini S., Pieraccini G., Lionetti P. (2010). Impact of diet in shaping gut microbiota revealed by a comparative study in children from Europe and rural Africa. Proc. Natl. Acad. Sci. USA.

[16] Stiemsma L.T., Arrieta M.C., Dimitriu P.A., Cheng J., Thorson L., Lefebvre D.L., Azad M.B., Subbarao P., Mandhane P., Becker A. (2016). Canadian Healthy Infant Longitudinal Development (CHILD) Study Investigators, Mohn WW, Finlay BB, Turvey SE. Shifts in *Lachnospira* and *Clostridium* sp. In the 3 month stool microbiome are associated with preschool age asthma. Clin. Sci. (Lond.).

[17] Stein M.M., Hrusch C.L., Gozdz J., Igartua C., Pivniouk V., Murray S.E., Ledford J.G., Marques dos Santos M., Anderson R.L., Metwali N. (2016). Innate immunity and asthma risk in Amish and Hutterite farm children. N. Engl. J. Med..

[18] Lee H.S., Park H.W., Song W.J., Jeon E.Y., Bang B., Shim E.J., Moon H.G., Kim Y.K., Kang H.R., Min K.U. (2016). TNF-α enhance Th2 and Th17 immune responses regulating by IL23 during sensitization in asthma model. Cytokine.

[19] Hongyan L. (2016). Esculetin attenuates Th2 and Th17 responses in an ovalbumin-induced asthmatic mouse model. Inflammation.

[20] McCoy K.D., Harris N.L., Diener P., Hatak S., Odermatt B., Hangartner L., Senn B.M., Marsland B.J., Geuking M.B., Hengartner H. (2006). Natural IgE production in the absence of MCH class II cognate help. Immunity.

[21] Cahenzli J., Köller Y., Wyss M., Geuking M.B., McCoy K.D. (2013). Intestinal microbial diversity during early life colonization shapes long-term IgE levels. Cell Host Microbe.

[22] Mazmanian S.K., Liu C.H., Tzianabos A.O., Kasper D.L. (2005). An immunomodulatory molecule of symbiotic bacteria directs maturation of the host immune system. Cell.

[23] Ivanov I.I., Atarashi K., Manel N., Brodie E.L., Shima T., Karaoz U., Wei D., Goldfarb K.C., Santee C.A., Lynch S.V. (2009). Induction of intestinal Th17 cells by segmented filamentous bacteria. Cell.

[24] Atarashi K., Tanoue T., Shima T., Imaoka A., Kuwahara T., Momose Y., Cheng G., Yamasaki S., Saito T., Ohba Y. (2011). Induction of colonic regulatory T cells by indigenous *Clostridium* species. Science.

[25] Orellano P., Quaranta N., Reynoso J., Balbi B., Vasquez J. (2017). Effect of outdoor air pollution on asthma exacerbations in children and adults: Systematic review and multilevel metanalysis. PLoS ONE.

[26] Hersoug L.G., Møller P., Loft S. (2016). Gut microbiota derived lipopolysaccharide uptake and trafficking to adipose tissue: Implications for inflammation and obesity. Obes. Rev..

[27] Dworkin J. (2014). The medium is the message: Interspecies and interkingdom signaling by peptidoglycan and related bacterial glycans. Annu. Rev. Microbiol..

[28] Pimentel M., Mathur R., Chang C. (2013). Gas and the microbiome. Curr. Gastroenterol. Rep..

[29] Furusawa Y., Obata Y., Fukuda S., Endo T.A., Nakato G., Takahashi D., Nakanishi Y., Uetake C., Kato K., Kato T. (2013). Commensal microbe-derived butyrate induces the differentiation of colonic regulatory T cells. Nature.

[30] Wannemuehler M.J., Kiyono H., Babb J.L., Michalek S.M., McGhee J.R. (1982). Lipopolysaccharide (LPS) regulation of the immune response: LPS converts germfree mice to sensitivity to oral tolerance induction. J. Immunol..

[31] Umbrello G., Esposito S. (2016). Microbiota and neurologic diseases: Potential effects of probiotics. J. Transl. Med..

[32] Principi N., Esposito S. (2016). Gut microbiota and central nervous system development. J. Infect..

[33] Yano J.M., Yu K., Donaldson G.P., Shastri G.G., Ann P., Ma L., Nagler C.R., Ismagilov R.F., Mazmanian S.K., Hsiao E.Y. (2015). Indigenous bacteria from the gut microbiota regulate host serotonin biosynthesis. Cell.

[34] Huang Y.J. (2015). The respiratory microbiome and innate immunity in asthma. Curr. Opin. Pulm. Med..

[35] Ascough S., Paterson S., Chiu C. (2018). Induction and subversion of human protective immunity: Contrasting influenza and respiratory syncytial virus. Front. Immunol..

[36] Dunphy-Doherty F., O’Mahony S.M., Peterson V.L., O’Sullivan O., Crispie F., Cotter P.D., Wigmore P., King M.V., Cryan J.F., Fone K.C.F. (2018). Post-weaning social isolation of rats leads to long-term disruption of the gut microbiota-immune-brain axis. Brain Behav. Immun..

[37] Elenius V., Palomares O., Waris M., Turunen R., Puhakka T., Rückert B., Vuorinen T., Allander T., Vahlberg T., Akdis M. (2017). The relationship of serum vitamins A, D, E and LL-37 levels with allergic status, tonsillar virus detection and immune response. PLoS ONE.

[38] Gauvreau G.M., O’Byrne P.M., Boulet L.P., Wang Y., Cockcroft D., Bigler J., FitzGerald J.M., Boedigheimer M., Davis B.E., Dias C. (2014). Effects of an anti-TSLP antibody on allergen-induced asthmatic responses. N. Engl. J. Med..

[39] Fahy J.V., Dickey B.F. (2010). Airway mucous function and dysfunction. N. Engl. J. Med..

[40] Roy M.G., Livraghi-Butrico A., Fletcher A.A., McElwee M.M., Evans S.E., Boerner R.M., Alexander S.N., Bellinghausen L.K., Song A.S., Petrova Y.M. (2014). MUC5B is required for airway defence. Nature.

[41] Staudinger B.J., Muller J.F., Halldórsson S., Boles B., Angermeyer A., Nguyen D., Rosen H., Baldursson O., Gottfreðsson M., Guðmundsson G.H., Singh P.K. (2014). Conditions associated with the cystic fibrosis defect promote chronic *Pseudomonas aeruginosa* infection. Am. J. Respir. Crit. Care Med..

[42] Barr J.J., Auro R., Furlan M., Whiteson K.L., Erb M.L., Pogliano J., Stotland A., Wolkowicz R., Cutting A.S., Doran K.S. (2013). Bacteriophage adhering to mucous provide a nonhost-derived immunity. Proc. Natl. Acad. Sci. USA.

[43] Van Helden M.J., Lambrecht B.N. (2013). Dendritic cells in asthma. Curr. Opin. Immunol..

[44] Kim T.H., Lee H.K. (2014). Differential roles of lung dendritic cell subsets against respiratory virus infection. Immune Netw..

[45] Backhed F., Ley R.E., Sonnenburg J.L., Peterson D.A., Gordon J.I. (2005). Host-bacterial mutualism in the human intestine. Science.

[46] Adams K.M., Lukas J., Kapur R.P., Stevens A.M. (2007). LPS induces traslocation of TLR4 in amniotic epiteliumin. Placenta.

[47] Aagaard K., Ma J., Antony K.M., Ganu R., Petrosino J., Versalovic J. (2014). The placenta harbors a unique microbiome. Sci. Transl. Med..

[48] Ardissone A.N., de la Cruz D.M., Davis-Richardson A.G., Rechcigl K.T., Li N., Drew J.C., Murgas-Torrazza R., Sharma R., Hudak M.L., Triplett E.W. (2014). Meconium microbiome analysis identifies bacteria correlated with premature birth. PLoS ONE.

[49] Munyaka P.M., Khafipour E., Ghia J.E. (2014). External influence of early childhood establishment of the gut microbiota and subsequent health implications. Front. Pediatr..

[50] Lai P.S., Kolde R., Franzosa E.A., Gaffin J.M., Baxi S.N., Sheehan W.J., Gold D.R., Gevers D., Xavier R.J., Phipatanakul W. (2018). The classroom microbiome and asthma morbidity in children attending 3 inner-city schools. J. Allergy Clin. Immunol..

[51] Penders J., Stobberingh E.E., van den Brandt P.A., Thijs C. (2007). The role of the intestinal microbiota in the development of atopic disorders. Allergy.

[52] Watson R.L., de Koff E.M., Bogaert D. (2018). Characterising the respiratory microbiome. Eur. Respir. J..

[53] Arrieta M.C., Sadarangani M., Brown E.M., Russell S.L., Nimmo M., Dean J., Turvey S.E., Chan E.S., Finlay B.B. (2016). A humanized microbiota mouse model of ovalbumin-induced lung inflammation. Gut Microbes.

[54] Vael C., Vanheirstraeten L., Desager K.N., Goossens H. (2011). Denaturing gradient gel electrophoresis of neonatal intestinal microbiota in relation to the development of asthma. BMC Microbiol..

[55] Penders J., Thijs C., van den Brandt P.A., Kummeling I., Snijders B., Stelma F., Adams H., van Ree R., Stobberingh E.E. (2007). Gut composition and development of atopic manifestation in infancy. Gut.

[56] Van Nimwegen F.A., Penders J., Stobberingh E.E., Postma D.S., Koppelman G.H., Kerkhof M., Reijmerink N.E., Dompeling E., van den Brandt P.A., Ferreira I. (2011). Mode and place of delivery, gastrointestinal microbiota, and their influence on asthma and atopy. J. Allergy Clin. Immunol..

[57] Björkstén B., Sepp E., Julge K., Voor T., Mikelsaar M. (2001). Allergy development and the intestinal microflora during the first year of life. J. Allergy Clin. Immunol..

[58] Schuijs M.J., Willart M.A., Vergote K., Gras D., Deswarte K., Ege M.J., Madeira F.B., Beyaert R., van Loo G., Bracher F. (2015). Farm dust and endotoxin protect against allergy through A20 induction in lung epithelial cell. Science.

[59] Dotterud C.K., Storrø O., Johnsen R., Oien T. (2010). Probiotics in pregnant women to prevent allergic disease: A randomized double blind trial. Br. J. Dermatol..

[60] Noverr M.C., Falkowski N.R., McDonald R.A., McKenzie A.N., Huffnagle G.B. (2005). Development of allergic airway disease in mice following antibiotic therapy and fungal microbiota increase: Role of host genetics, antigen, and interleukin-13. Infect. Immun..

[61] Noverr M.C., Noggle R.M., Toews G.B., Huffnagle G.B. (2004). Role of antibiotics and fungal microbiota in driving pulmonary allergic responses. Infect. Immun..

[62] Hunt J.R., Martinelli R., Adams V.C., Rook G.A., Brunet L.R. (2005). Intragastric administration of *Mycobacterium vaccae* inhibits severe pulmonary allergic inflammation in a mouse model. Clin. Exp. Allergy.

[63] Arnold I.C., Dehzad N., Reuter S., Martin H., Becher B., Taube C., Muller A. (2011). *Helicobacter pylori* infection prevents allergic asthma in mousemodels through the induction of regulatory T cells. J. Clin. Investig..

[64] Forsythe P., Inman M.D., Bienenstock J. (2007). Oral treatment with live Lactobacillus reuteri inhibits the allergic airway response in mice. Am. J. Respir. Crit. Care Med..

[65] Lyons A., O’Mahony D., O’Brien F., MacSharry J., Sheil B., Ceddia M., Russell W.M., Forsythe P., Bienenstock J., Kiely B. (2010). Bacterial strain-specific induction of Foxp3+T regulatory cells is protective in murine allergy models. Clin. Exp. Allergy.

[66] Josefowicz S.Z., Niec R.E., Kim H.Y., Treuting P., Chinen T., Zheng Y., Umetsu D.T., Rudensky A.Y. (2012). Extrathymically generated regulatory T cells control mucosal Th2 inflammation. Nature.

[67] Ngoc P.L., Gold D.R., Tzianabos A.O., Weiss S.T., Celedón J.C. (2005). Cytokines, allergy, and asthma. Curr. Opin. Allergy Clin. Immunol..

[68] Kusel M.M., de Klerk N.H., Kebadze T., Vohma V., Holt P.G., Johnston S.L., Sly P.D. (2007). Early-life respiratory viral infections, atopic sensitization, and risk of subsequent development of persistent asthma. J. Allergy Clin. Immunol..

[69] Wu P., Hartert T.V. (2011). Evidence for a causal relationship between respiratory syncytial virus infection and asthma. Expert Rev. Anti-Infect. Ther..

[70] Esposito S., Principi N. (2017). Impact of nasopharyngeal microbiota on the development of respiratory tract diseases. Eur. J. Clin. Microbiol. Infect. Dis..

[71] Bisgaard H., Hermansen M.N., Buchvald F., Halkjaer L.B., Bønnelykke K., Brasholt M., Heltberg A., Vissing N.H., Thorsen S.V., Stage M. (2007). Childhood asthma after bacterial colonization of the airway in neonates. N. Engl. J. Med..

[72] Holt P.G., Strickland D.H., Hales B.J., Sly P.D. (2014). Defective respiratory tract immune surveillance in asthma: A primary causal factor in disease onset and progression. Chest.

[73] Ege M.J., Mayer M., Normand A.C., Genuneit J., Cookson W.O., Braun-Fahrländer C., Heederik D., Piarroux R., von Mutius E. (2011). GABRIELA Transregio 22 Study Group: Exposure to environmental microorganisms and childhood asthma. N. Engl. J. Med..

[74] Fazlollahi M., Lee T.D., Andrade J., Oguntuyo K., Chun Y., Grishina G., Grishin A., Bunyavanich S. (2018). The nasal microbiome in asthma. J. Allergy Clin. Immunol..

[75] Stokholm J., Blaser M.J., Thorsen J., Rasmussen M.A., Waage J., Vinding R.K., Schoos A.M., Kunøe A., Fink N.R., Chawes B.L. (2018). Maturation of the gut microbiome and risk of asthma in childhood. Nat. Commun..

[76] Denning D.W., O’Driscoll B.R., Hogaboam C.M., Bowyer P., Niven R.M. (2006). The link between fungi and severe asthma: A summary of the evidence. Eur. Respir. J..

[77] Mennini M., Dahdah L., Artesani M.C., Fiocchi A., Martelli A. (2017). Probiotics in asthma and allergy prevention. Front. Pediatr..

[78] Zelante T., Pieraccini G., Scaringi L., Aversa F., Romani L. (2016). Learning from other diseases: Protection and pathology in chronic fungal infections. Semin. Immunopathol..

[79] Wheeler M.L., Limon J.J., Bar A.S., Leal C.A., Gargus M., Tang J., Brown J., Funari V.A., Wang H.L., Crother T.R. (2016). Immunological consequences of intestinal fungal dysbiosis. Cell Host Microbe.

[80] Mao K., Baptista A.P., Tamoutounour S., Zhuang L., Bouladoux N., Martins A.J., Huang Y., Gerner M.Y., Belkaid Y., Germain R.N. (2018). Innate and adaptive lymphocytes sequentially shape the gut microbiota and lipid metabolism. Nature.

[81] Germain R.N., Huang Y. (2018). ILC2s—Resident lymphocytes pre-adapted to a specific tissue or migratory effectors that adapt to where they move?. Curr. Opin. Immunol..

[82] Huang Y., Mao K., Chen X., Sun M.A., Kawabe T., Li W., Usher N., Zhu J., Urban J.F., Paul W.E. (2018). S1P-dependent interorgan trafficking of group 2 innate lymphoid cells supports host defense. Science.

[83] Ekmekciu I., von Klitzing E., Neumann C., Bacher P., Scheffold A., Bereswill S., Heimesaat M.M. (2017). Fecal microbiota transplantation, commensal *Escherichia coli* and *Lactobacillus johnsonii* strains differentially restore intestinal and systemic adaptive immune cell populations following broad-spectrum antibiotic treatment. Front. Microbiol..

[84] Durack J., Lynch S.V. (2018). The gut microbiome: Relationships with disease and opportunities for therapy. J. Exp. Med..

[85] Turturice B.A., McGee H.S., Oliver B., Baraket M., Nguyen B.T., Ascoli C., Ranjan R., Rani A., Perkins D.L., Finn P.W. (2017). Atopic asthmatic immune phenotypes associated with airway microbiota and airway obstruction. PLoS ONE.

[86] Durack J., Lynch S.V., Nariya S., Bhakta N.R., Beigelman A., Castro M., Dyer A.M., Israel E., Kraft M., Martin R.J. (2017). National Heart, Lung and Blood Institute’s “AsthmaNet”: Features of the bronchial bacterial microbiome associated with atopy, asthma, and responsiveness to inhaled corticosteroid treatment. J. Allergy Clin. Immunol..

[87] Ozturk A.B., Turturice B.A., Perkins D.L., Finn P.W. (2017). The potential for emerging microbiome-mediated therapeutics in asthma. Curr. Allergy Asthma Rep..

[88] Durack J., Kimes N.E., Lin D.L., Rauch M., McKean M., McCauley K., Panzer A.R., Mar J.S., Cabana M.D., Lynch S.V. (2018). Delayed gut microbiota development in high-risk for asthma infants is temporarily modifiable by *Lactobacillus* supplementation. Nat. Commun..

